# 8-Hydroxydeoxyguanosine: a new potential independent prognostic factor in breast cancer

**DOI:** 10.1038/sj.bjc.6605565

**Published:** 2010-02-23

**Authors:** H Sova, A Jukkola-Vuorinen, U Puistola, S Kauppila, P Karihtala

**Affiliations:** 1 Department of Oncology and Radiotherapy, Oulu University Hospital, P.O. Box 22, FIN-90029, Finland; 2 Department of Obstetrics and Gynecology, Oulu University Hospital, P.O. Box 24, FIN-90029, Finland; 3 Department of Pathology, Oulu University Hospital, P.O. Box 50, FIN-90029, Finland

**Keywords:** 8-oxodG, DNA repair, immunohistochemistry, oxidative stress, reactive oxygen species

## Abstract

**Background:**

8-Hydroxydeoxyguanosine (8-oxodG) is the commonly used marker of oxidative stress-derived DNA damage. 8-OxodG formation is regulated by local antioxidant capacity and DNA repair enzyme activity. Earlier studies have reported contradictory data on the function of 8-oxodG as a prognostic factor in different cancer types.

**Methods:**

We assessed pre-operative serum 8-oxodG levels with an enzyme-linked immunosorbent assay in a well-defined series of 173 breast cancer patients. 8-OxodG expression in the nuclei of cancer cells from 150 of these patients was examined by immunohistochemistry.

**Results:**

The serum 8-oxodG levels and immunohistochemical 8-oxodG expression were in concordance with each other (*P*<0.05). Negative 8-oxodG immunostaining was an independent prognostic factor for poor breast cancer-specific survival according to the multivariate analysis (*P*<0.01). This observation was even more remarkable when ductal carcinomas only (*n*=140) were considered (*P*<0.001). A low serum 8-oxodG level was associated statistically significantly with lymphatic vessel invasion and a positive lymph node status.

**Conclusions:**

Low serum 8-oxodG levels and a low immunohistochemical 8-oxodG expression were associated with an aggressive breast cancer phenotype. In addition, negative 8-oxodG immunostaining was a powerful prognostic factor for breast cancer-specific death in breast carcinoma patients.

Reactive oxygen species (ROS), despite being products of normal cellular metabolism, are considered to have a substantial influence on the development of cancer, in part, because of their ability to react with DNA. For example hydroxyl radical (^•^OH) can react with pyrimidines, purines and chromatin protein resulting in base modifications, genomic instability and alterations in gene expression. These reactions in connection with oncogenes or tumour suppressor genes may result in the initiation of cancer (Loft and Poulsen, 1996). Several recent studies have shown high ROS levels in carcinoma cells compared with the surrounding healthy tissue (reviewed in Karihtala and Soini, 2007). Under normal conditions, ROS are maintained within narrow boundaries by scavenging systems, such as superoxide dismutases, peroxiredoxins (Prx) and glutathione-related antioxidant defences. Consequently, when the amount of ROS exceeds the capacity of the ROS scavenging systems, oxidative stress occurs and this imbalanced redox status leads to an increase in damage to DNA.

A direct measurement of ROS is challenging because of their short lifetime and immediate reaction with redox state regulating components. For instance, it has been estimated that the lifespan of ^•^OH, the most harmful ROS, is <1 ns (Valko *et al*, 2004). Therefore, a useful method to assess ROS is the use of antibodies against the specific ‘footprints’ of oxidative damage. 8-Hydroxydeoxyguanosine (8-oxodG) is a specific marker of 2′-deoxyguanosine damage after ROS attack to DNA. 8-OxodG is one of the most widely used oxidative stress biomarkers, and it can be measured with immunohistochemistry and, for example by enzyme-linked immunosorbent assay (ELISA) or high pressure liquid chromatography, with mass spectrometric or electrochemical detection (HPLC-MS/MS; HPLC-EC) in serum or urine samples (Cooke *et al*, 2008). There are currently no data as to whether systemic 8-oxodG levels are associated with 8-oxodG modifications *in situ* in any disease or whether serum or immunohistochemical assessment of 8-oxodG could be used as a prognostic factor in breast cancer.

In this study, we analysed serum 8-oxodG levels and 8-oxodG tissue expression from breast carcinoma patients and correlated the results with clinicopathological parameters such as the stage, grade and lymphatic and blood vessel invasion status. The function of 8-oxodG as a prognostic factor in breast cancer and correlation between serum 8-oxodG levels and 8-oxodG breast carcinoma tissue expression was evaluated.

## Materials and methods

The study material consisted of 173 pre-operative venous blood samples from breast carcinoma patients, which were acquired from the files of the Department of Oncology, Oulu University Hospital from 2003 to 2005. In addition, we were able to acquire 150 out of 173 tumour blocks from these patients for immunohistochemical analysis. The tumour blocks were collected from the archives of the Department of Pathology, Oulu University Hospital. The study was approved by the Local Ethics Committee.

Blood samples were taken before primary operations and serum samples were stored in polypropylene or polystyrene tubes at −80°C until the time of analysis. The breast cancer tissue samples were fixed in neutral formalin and embedded in paraffin. The malignancy grades in the cancerous lesions were determined according to the WHO classification (Tavassoli and Devilee, 2003) by pathologist (SK). The material comprised 140 ductal carcinomas, 25 lobular carcinomas and 8 other types of breast carcinomas. The clinical data were sourced from the records of Oulu University Hospital. The most important patient and tumour characteristics are shown in Table 1. The mean follow-up time of the subjects was 40.5 months.

The serum levels of 8-oxodG were determined using an ELISA using the Highly Sensitive 8-OHdG kit, which was obtained from the Japan Institute for the Control of Aging, Fukuroi, Japan. The kit uses an anti 8-oxodG monoclonal antibody (clone N45.1), which is highly specific for 8-oxodG. The ELISA assay was performed according to the manufacturer's instructions with a few divergences. At first, we pre-processed all serum samples using Millipore Microcon filters. Filters were damped with 100 *μ*l of distilled water and subsequently centrifuged in 14.000 **g** for 5 min. Then filters were turned around and centrifuged for another 5 min to remove remaining water. Damped filters were moved to new tubes and 200 *μ*l of serum sample was added into each tube and then centrifuged for 30 min in 14.000 **g**. Next, the primary antibody was reconstituted with the primary antibody solution. Then 50 *μ*l of sample or standard were added to wells, doing duplicate for each. After that, 50 *μ*l of reconstituted primary antibody was added to each well. Plate was shaken and covered with adhesive strip and then incubated at 4°C for over night. After incubation, the contents of the wells were poured off and each well was washed with 250 *μ*l of washing solution three times. Then secondary antibody was reconstituted with the secondary antibody solution; 100 *μ*l of constituted secondary antibody was added to each well. Next, the plate was shaken, covered with adhesive strip and then incubated for 1 h at room temperature. Washing was repeated at the end of the incubation period. After that, a substrate solution was prepared and 100 *μ*l of it was added to each well and the plate was shook. The plate was incubated in the dark for 15 min at room temperature. Then 100 *μ*l of reaction terminating solution was added to each well and the plate was shook. Absorbances were measured at 450 nm in a plate reader and standard curve was used to determine the amount of 8-oxodG in samples. We assayed the duplicates of each sample and used also the outermost wells of the microtiter plate. With this method, we were able to assay 41 samples in one plate. The 8-oxodG concentrations from the duplicate samples were extremely close to each other throughout the analysis, and if they differed to >10%, they were assayed again. Four out of 173 samples were re-assayed because of over 10% variance between duplicates.

For immunohistochemistry, the paraffin-embedded breast lesions were first sectioned on slides of 4 *μ*m thickness and placed on SuperFrostPlus glass (Menzel–Glāser, Germany). To remove the paraffin, they were soaked in xylene and then rehydrated in a graded alcohol series. They were heated in a microwave oven in 10 mm of citric acid monohydrate for 10 min to predigest the sections, and then chilled at room temperature. The sections were immersed in 3% hydrogen peroxide in methanol for 15 min to consume the endogenous peroxide. The slides were incubated with a 1 : 125 primary antibody dilution against 8-oxodG (Mouse monoclonal 8-oxodG antibody, Gentaur, Belgium) overnight at +4°C. Both ELISA kit and immunostainings were based on the same 8-oxodG antibody (N45.1) (Toyokuni *et al*, 1997). Immunostaining was carried out by using a biotinylated secondary antibody 1 : 400 dilution with an avidin–biotin–peroxidase complex (Dakopatts, Glostrup, Denmark). Aminoethyl carbazole (Zymed Laboratories Inc., South San Francisco, California, USA) was used as a chromogen. Immersed in 2% ammonia water, Meyer's haematoxylin was used for counterstaining, and in the end, the sections were mounted with Immu-Mount (Shandon, Pittsburgh, PA, USA).

The intensity of the 8-oxodG immunostainings from the nuclei of cancer cells was evaluated by dividing the staining reaction into four groups: −=negative immunostaining (<5% of tumour cells showing nuclear positivity), +=weak immunostaining (5–20% of tumour cells showing nuclear positivity), ++=moderate immunostaining (21–80% of tumour cells showing nuclear positivity) and +++=strong immunostaining (>80% of tumour cells showing nuclear positivity). For statistical analysis, we divided the immunostaining results into two groups: negative (−) and positive (+, ++, +++) immunostaining. The distribution of the immunostaining groups is shown in Table 2.

SPSS 15.0 for Windows and R-language were used for statistical analysis. The significance of the associations was defined using the Spearman's test, Mann–Whitney *U*-test and Pearson *χ*^2^-test with Fisher's two-sided exact test for 8-oxodG ELISA and 8-oxodG immunohistochemistry, respectively. Survival was analysed with the Kaplan–Meier curve with log-rank, Tarone–Ware and Breslow tests. Cox multivariate regression analysis was used for multivariate analysis. The probability values <0.05 were considered significant. We also cooperated with a statistics expert as needed.

## Results

The 8-oxodG serum levels and tissue expression of the whole patient group, and separately of the patients with ductal carcinoma, were compared with several known tumour characteristics. The presence of lymphatic invasion, blood vessel invasion, lymph node metastases, expression of oestrogen receptor, progesterone receptor, HER-2, Ki-67 and p53 were classified as either positive or negative for statistical analyses. The grading and tumour size were divided into the following subgroups: grade I group, grade II–III group, T1 group and T2–4 group.

When the characteristics were compared within the whole patient group, the lower serum 8-oxodG levels were associated with lymphatic vessel invasion (*P*<0.05) and positive lymph node status (*P*<0.05) (Table 3). Among the ductal carcinoma patients, similarly low serum 8-oxodG levels correlated with poor differentiation (*P*<0.05), lymphatic and blood vessel invasions (*P*<0.05) and node status (*P*<0.01). Serum 8-oxodG was not a statistically significant prognostic factor in either the whole or the ductal cancer patient groups.

**Figure 1:**
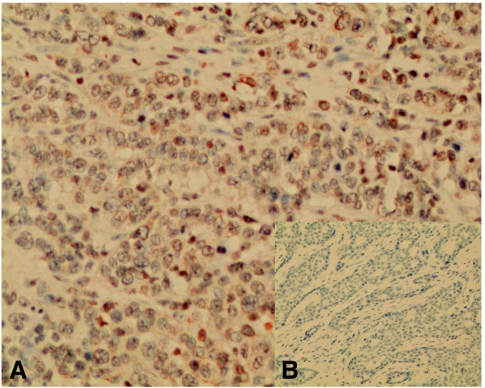
An example of highly positive (**A**) (magnification × 210) and negative (**B**) (magnification × 105) 8-oxodG immunostaining.

The 8-oxodG immunostaining localised subcellularly to nuclei (Figure 1). Negative 8-oxodG immunostaining correlated with negative HER-2 (*P*<0.05) and p53 (*P*<0.05) expression within the whole study group. In addition, negative 8-oxodG immunostaining was associated with positive node status among ductal carcinoma patients (*P*<0.05). The 8-oxodG levels from serum and 8-oxodG tissue expression correlated positively with each other according to the Spearman's test (*P*<0.05, correlation coefficient 0.163). Neither serum nor immunohistochemical 8-oxodG were associated with the patient age or menopausal status.

**Figure 2:**
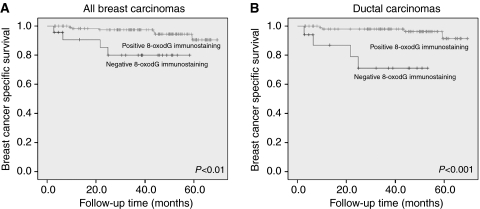
Kaplan–Meier curves showing breast cancer-specific survival when immunohistochemical 8-oxodG staining is divided into either negative or positive: (**A**) all carcinomas (log-rank *P*<0.01) and (**B**) ductal carcinomas only (log-rank *P*<0.001).

Patients with negative 8-oxodG immunostaining had a higher risk of breast cancer-specific death compared with patients with positive 8-oxodG immunostaining (Kaplan–Meier, log-rank analysis *P*<0.01; Figure 2). Survival statistics are shown in Table 4. In the Cox multivariate analysis, a negative 8-oxodG immunohistochemistry was an independent prognostic factor of poor survival when tumour size, node status, grade, Ki-67, HER-2, p53 and receptor status were taken into account.

## Discussion

In this study, we show for the first time that negative 8-oxodG immunostaining in breast cancer cells is an independent prognostic factor for poorer prognosis and that low serum and tissue levels of 8-oxodG are characteristic of more aggressive breast cancer. 8-OxodG is one of the most widely used biomarkers of oxidative stress, mainly because of its abundance in DNA and also because of its reliable detectability (Karihtala and Soini, 2007). Compared with other bases, guanine most readily undergoes an oxidative attack in the presence of ROS (Peoples and Karnes, 2005). 8-OxodG can induce GC → TA transversion mutations particularly during DNA replication and thus, if these oxidative lesions are not repaired, they can become mutagenic (Peoples and Karnes, 2005). There is a rather broad consensus that extracellular 8-oxodG levels are not affected by diet, cell death or artefactual formation (Cooke *et al*, 2005). However, this is the first study to show that oxidative stress observed in cancer cells reflects a good correlation to the serum 8-oxodG levels. Therefore, it seems that serum 8-oxodG in breast cancer patients mainly originates from tumour tissue.

Earlier, many studies examining oxidatively damaged DNA in carcinogenesis have discovered augmented 8-oxodG levels in either the urine or tumour tissue DNA compared with the healthy tissue. For example, in studies on breast carcinomas, the 8-oxodG levels have been reported as being 8 to 17 times higher in comparison with those in healthy breast tissue (Matsui *et al*, 2000). Elevated levels of 8-oxodG from cancer patients compared with healthy subjects have also been observed in lung cancer (Vulimiri *et al*, 2000; Shen *et al*, 2007), basal cell carcinoma (Nishigori *et al*, 2005), bladder cancer (Kaczmarek *et al*, 2005), acute lymphoblastic leukaemia (Sentürker *et al*, 1997), colorectal cancer (Oliva *et al*, 1997), high grade cervical dysplasia (Romano *et al* 2000), renal cell carcinoma (Okamoto *et al*, 1994), prostate cancer (Miyake *et al*, 2004), gastric intestinal metaplasia (Farinati *et al*, 2008) and gastric adenocarcinoma (Lee *et al*, 1998). Evidence from these studies suggests that elevated 8-oxodG levels in these malignant or premalignant diseases compared with healthy individuals would be a sign of increased oxidative stress, impaired antioxidant defence or inadequate repair of oxidatively damaged DNA. A recent study showed a highly significant decrease of 8-oxodG serum levels after breast cancer surgery compared with pre-operative levels (Cho *et al*, 2009). However, there are also some reports that have found no difference in the 8-oxodG levels between cancer patients and healthy subjects (Nagashima *et al*, 1995; Jałoszyñski *et al*, 2003).

In this study, low immunohistochemical expression and low pre-operative serum 8-oxodG levels were strongly associated with conventional prognostic factors for aggressive breast cancer such as positive lymph node status and lymphovascular invasion. This significant association was even more obvious among ductal carcinomas, which is the main histological subtype of breast cancer with a highly variable prognosis and, therefore, more accurate prognostic factors are needed especially for this histological breast cancer subtype. In parallel with this, the presence of 8-oxodG immunostaining correlated with positive HER-2 status and high p53 expression. HER-2 is a one of the most widely used tumour markers in breast cancer, and HER-2 gene amplification results in poor prognosis, resistance to hormonal therapies and generally to more aggressive breast cancer phenotype (Slamon *et al*, 1987; Ross *et al*, 2009). p53 is a tumour suppressor protein, whose physiological function is highly essential in safeguarding DNA integrity (Barnes and Camplejohn, 1996). The co-expressions between 8-oxodG and p53 and HER-2 are probably because of the nature of 8-oxodG as a marker of oxidative stress-derived DNA mutations in general and suggest that ROS has a function in the formation of HER-2 and p53 mutations. Earlier, ROS-derived GC → TA transversions have been observed in the p53 gene *in vivo* in lung and liver cancers (Hussain *et al*, 2000; Cooke *et al*, 2006).

Furthermore, according to our results, negative 8-oxodG immunostaining from tumour tissue could be used as an independent prognostic factor for poor breast cancer-specific survival. Earlier, two groups have reported that low 8-oxodG levels either in tumour tissue or in urine of breast cancer patients are associated with a higher stage or grade (Matsui *et al*, 2000; Kuo *et al*, 2007). In both of these studies, 8-oxodG levels were elevated in cancer patients compared with healthy controls. However, these studies were smaller than ours and no associations between 8-oxodG and survival were reported. In a study by Dincer *et al* (2007), the 8-oxodG plasma levels were significantly higher in controls than in gastric and colon carcinoma patients, which is also in line with our results. However, high 8-oxodG levels from tumour tissue DNA have also been reported as an independent prognostic factor of poor survival in lung cancer and hepatocellular carcinoma (Matsumoto *et al*, 2003; Shen *et al*, 2007). This emphasizes the diverse prognostic function of 8-oxodG in different malignancies.

There are several possible mechanisms behind the inverse association of 8-oxodG levels and tumour aggressiveness. Low serum, plasma or urine levels of 8-oxodG can be a sign of enfeebled repair of oxidatively damaged DNA or enhanced antioxidant defence rather than low ROS production. The main repair enzyme for 8-oxodG is human 8-oxoguanine DNA glycosylase 1 (hOGG1) and its proper function is crucial for the prevention of G to T transversion mutations (Hirano, 2008). Reduced hOGG1 levels significantly increase relative risk for initiation of carcinomas (Paz-Elizur *et al*, 2003, 2006). With impaired hOGG1 function, cells are not able to cleave damaged guanosine from DNA, which results in lower 8-oxodG levels in extracellular fluids. However, defects in DNA repair do not explain low 8-oxodG expression in the tumour tissue of the most aggressive breast carcinomas. High ROS production in tumour tissue promotes the over-expression of antioxidant proteins, such as thioredoxins and Prx, which are associated with malignant transformation in breast cancer (Karihtala *et al*, 2003; Turunen *et al*, 2004). In addition, promoted antioxidant defence in tumour tissue could offer a growth advantage to cancer cells by avoiding apoptosis and necrosis caused by ROS. Overproduced antioxidant enzymes would prevent ROS interaction with DNA leading to decreased formation of 8-oxodG at tissue level as suggested by the current results. Transcription factor NF-E2-related factor 2 (Nrf2), the major up-regulator of multiple antioxidant enzymes (e.g. peroxiredoxin I, thioredoxin reductase), has a highly important function in eliminating ROS from the cells. On the other hand, Nrf2 up-regulation, which is commonly seen in chemoresistant cell lines, may provide growth advantage to cancer cells during oncological treatments (Lau *et al*, 2008, Singh *et al*, 2008). Although the clinical data is currently lacking from breast cancer patients, Nrf2 up-regulation and consequent antioxidant enzyme induction and chemoresistance may explain why the patients with the worst prognosis have low 8-oxodG levels at the initial situation.

The function of oxidative stress has also been studied in some non-malignant diseases (Evans and Cooke, 2006). Interestingly, low levels of 8-oxodG have been reported in the urine of systemic lupus erythematosus patients and in cerebrospinal fluid of patients with Alzheimer's disease when compared with healthy subjects (Evans and Cooke, 2006; Farinati *et al*, 2008). These authors also concluded that low 8-oxodG levels should be taken as evidence of impaired DNA repair of 8-oxodG.

We conclude that immunohistochemical 8-oxodG expression is associated with the serum 8-oxodG levels among breast cancer patients. Low 8-oxodG levels both in serum and in breast cancer cells strongly indicate a more aggressive disease, especially in ductal carcinomas. Negative 8-oxodG immunohistochemical staining is a powerful prognostic factor in breast carcinoma patients. The mechanism behind these results offers an attractive topic for future studies.

## Figures and Tables

**Table 1:** Patient and tumour characteristics

	**Ductal carcinoma (*n*=140)**	**Lobular carcinoma (*n*=25)**	**Other carcinomas (*n*=8)**	**Total (*n*=173)**
*Average age (years)*	56.4±12.1	58.2±16.7	66.5±13.3	57.1±13.0
*Grade*				
I	18 (12.9%)	2 (8%)	7 (87.5%)	26 (15.6%)
II	64 (45.7%)	19 (76%)	1 (12.5%)	83 (48.6%)
III	58 (41.4%)	4 (16%)	0 (0%)	62 (35.8%)
				
*T*				
1	89 (63.6%)	16 (64%)	5 (62.5%)	110 (63.6%)
2	44 (31.4%)	8 (32%)	3 (37.5%)	55 (31.8%)
3+4	7 (5%)	1 (4%)	0 (0%)	8 (4.7%)
				
*N*				
0	75 (53.6%)	14 (56%)	8 (100%)	97 (56.1%)
1	58 (41.4%)	8 (32%)	0 (0%)	66 (38.2%)
2	7 (5%)	3 (12%)	0 (0%)	10 (5.8%)
				
*HER-2*				
Positive	20 (14.3%)	2 (8%)	0 (0%)	22 (12.7%)
Negative	120 (85.7%)	23 (92%)	8 (100%)	151 (87.3%)
				
*Estrogen receptor*				
Positive	107 (76.4%)	25 (100%)	8 (100%)	140 (80.9%)
Negative	33 (23.6%)	0 (0%)	0 (0%)	33 (19.1%)
				
*Progesterone receptor*				
Positive	90 (64.3%)	19 (76%)	7 (87.5%)	116 (67.1%)
Negative	50 (35.7%)	6 (24%)	1 (12.5%)	57 (32.9%)
				
*Lymphatic vessel invasion*				
Yes	15 (10.7%)	0 (0%)	1 (12.5%)	16 (9.2%)
No	119 (85.0%)	25 (100%)	7 (87.5%)	151 (87.3%)
Unknown	6 (4.3%)			6 (3.5%)
				
*Blood vessel invasion*				
Yes	9 (6.4%)	0 (0%)	0 (0%)	9 (5.2%)
No	125 (89.3%)	25 (100%)	8 (100%)	158 (91.3%)
Unknown	6 (4.3%)			6 (3.5%)
				
*Treated with*				
Radiotherapy	131 (93.6%)	23 (92%)	4 (50%)	158 (91.3%)
Chemotherapy	75 (53.6%)	7 (28%)	0 (0%)	82 (47.4%)
Hormonal therapy	60 (42.9%)	10 (40%)	0 (0%)	70 (40.5%)

**Table 2:** Distribution of 8-oxodG immunostaining

	**All patients (*n*=150)**	**Ductal carcinomas (*n*=123)**
−	23 (15.3%)	17 (13.8%)
+	23 (15.3%)	23 (18.7%)
++	64 (42.7%)	53 (43.1%)
+++	40 (26.7%)	30 (24.4%)

Abbreviation: 8-oxodG=8-hydroxydeoxyguanosine.

**Table 3:** Mean serum 8-oxodG levels and s.d. in the groups of conventional prognostic factors

	**Mean 8-oxodG (ng ml^–1^)**	***P*-value**
*Tumor size (T)*		
1	0.18±0.13	NS
2–4	0.15±0.11	
		
*Nodal status (N)*		
0	0.18±0.12	*P*<0.05
1–2	0.15±0.13	
		
*Grade*		
1–2	0.17±0.12	NS
3	0.17±0.13	
		
*Lymphatic vessel invasion*		
Yes	0.12±0.09	*P*<0.05
No	0.17±0.13	
		
*Blood vessel invasion*		
Yes	0.10±0.04	NS
No	0.17±0.13	
		
*ER expression*		
Positive	0.16±0.12	NS
Negative	0.19±0.14	
		
*PR expression*		
Positive	0.16±0.12	NS
Negative	0.19±0.13	
		
*Ki-67*		
0–2	0.16±0.12	NS
3	0.19±0.14	
		
*Her-2*		
Positive	0.15±0.09	NS
Negative	0.17±0.13	
		
*Histology*		
Ductal	0.17±0.13	NS
Other	0.16±0.13	

Abbreviations: ER=oestrogen receptor; NS=no statistical significance; PR=progesterone receptor; 8-oxodG=8-hydroxydeoxyguanosine.

**Table 4:** Survival statistics for 8-oxodG immunohistochemistry and conventional risk factors

	**Patients (*n*=173)**	**Mean survival (months)**	***P*-value for BCSS**
*Tumor size (T)*			
1	110	68.7	<0.01
2–4	63	63.5	
			
*Nodal status (N)*			
0	97	70.4	<0.01
1–2	76	62.6	
			
*Grade*			
1–2	110	70.0	<0.01
3	63	62.1	
			
*Lymphatic vessel invasion*			
Yes	16	55.6	NS
No	151	67.9	
			
*Blood vessel invasion*			
Yes	9	58.2	NS
No	158	67.6	
			
*ER expression*			
Positive	140	69.0	<0.01
Negative	33	55.9	
			
*PR expression*			
Positive	116	67.5	<0.05
Negative	57	64.3	
			
*Ki-67*			
0–2	127	69.6	<0.001
3	46	56.7	
			
*Her-2*			
Positive	22	60.8	NS
Negative	151	67.6	
			
*Histology*			
Ductal	140	66.4	NS
Other	33	67.7	
			
*8-oxodG immunohistochemistry*			
Positive	127	66.9	<0.01
Negative	23	49.5	
			
*8-oxodG immunohistochemistry ductal histology*			
Positive	106	67.4	<0.001
Negative	17	42.1	

Abbreviations: BCSS=Breast cancer specific survival; ER=oestrogen receptor; NS=no statistical significance; PR=progesterone receptor; 8-oxodG=8-hydroxydeoxyguanosine.
